# Conference Report: WORKSHOP ON CHARACTERIZING DIAMOND FILMS IV Gaithersburg, MD March 4–5, 1996

**DOI:** 10.6028/jres.101.042

**Published:** 1996

**Authors:** Albert Feldman, Ajay P. Malshe, John E. Graebner

**Affiliations:** National Institute of Standards and Technology, Gaithersburg, MD 20899-0001; University of Arkansas, Fayetteville, AR 72701; Lucent Technologies, Murray Hill, NJ 07974

## 1. Introduction and Conclusions

The fourth in a series of workshops was held at NIST on March 4–5, 1996 to discuss, in depth, specific topics deemed important to the characterization of diamond films made by chemical vapor deposition (CVD diamond) and to address the need for standards in diamond technology. There were 28 registered participants at the workshop.

We focussed on two technical topics for discussion: adhesion of CVD diamond to cutting tool materials and standardization of thermal conductivity measurements for heat spreading applications.

The principal conclusions of the workshop include:

- Considerable progress has been made in the development of diamond coatings that adhere adequately to tungsten carbide (WC) cutting tool substrates. Some expressed the view that the adhesion problem has been solved as attested to by the availability of off-the-shelf diamond coated cutting tool inserts.
- To promote the use of diamond coated cutting tools, protocols for their use will have to be developed. However, a considerable effort would be required due to the large range of machining conditions that can be encountered.
- The reasons for the relatively large laboratory-to-laboratory variations in our prior round robin on thermal conductivity measurement can be attributed to specimen inhomogeneity and specimen anisotropy. A new round robin based on specimens with significantly better homogeneity is being organized.

## 2. Adhesion of CVD Diamond to Cutting Tool Materials

This symposium consisted of 11 speakers distributed into 4 sessions.

The first session of the workshop consisted of three speakers. E. Sevillano of ASTeX gave a review of adhesion technology. He expressed the opinion that the adhesion problem had been solved citing as evidence the availability of carbide cutting tools coated with diamond. C. A. Klein of c.a.k. analytics described some aspects of mechanical modeling. C. H. Shen of General Motors provided considerable insight into the diamond coating issues of tooling. He expressed the view that adhesion of diamond coated cemented carbide inserts appears to be sufficient. However, our current understanding about the role of diamond microstructure on machining performance is limited. Additional effort is needed to optimize the diamond film properties in order to minimize abrasive wear.

In the second session, W. Schmidt of Auburn University presented some preliminary results on measuring thickness and relating adhesion of the diamond film to x-ray measurements of stress at the diamond-carbide interface. The approach compares x-ray diffraction from an uncoated carbide substrate with diffraction from coated inserts with different thicknesses of the diamond films and as a function of position on the insert. Another technique, the ac-magnetic bridge, shows promise as a nondestructive method of measuring the thickness of a deposited diamond layer. Preliminary results show some correlation between the thicknesses measured by the magnetic bridge method and thickness measurements made with a scanning electron microscope (SEM). Refinement of the magnetic bridge technique, such as reduction in the size of the magnetic probe, will enable the thickness to be measured over the surface of the insert.

J. Zimmer of sp3 Inc. opened his presentation with an analysis of factors affecting the deposition of diamond on cemented carbide and adhesion of the diamond film. He emphasized that both bulk and surface properties of the carbide and the diamond will affect adhesion, as well as the interface between the two. Of particular importance are stress levels, surface topography, nucleation density, carbide substrate properties, such as surface composition, bulk expansion coefficient, stiffness, surface roughness and contamination with cobalt, tungsten oxide, brittle ETA-phases and loose carbide grains. He pointed out the need for improved quantitative accelerated tests of the coated tools in a laboratory environment.

P. Misty of QQC Inc. presented recent advances in multiplexed laser technology for surface property modification and diamond deposition. The multiplexed laser system is based on a combination of 3 to 4 pulsed laser beams of widely different wavelengths swept over the entire surface of the object to be coated. Substrate materials used were carbides, cermets, ceramics, and high speed steels. Diamond coatings ~40 μm thick were deposited on inserts at a rate of 45 s per insert. Finally, he discussed current and potential capabilities for the diamond coatings market, emphasizing diamond coated cemented carbide inserts and tools.

In the third session, A. Inspektor of Kennametal discussed his views on adhesion of diamond to WC-Co (tungsten carbide with a cobalt binder) cutting tools His presentation was directed primarily toward the substrate surface structure and the need to produce a rough, cobalt free surface to achieve good adhesion. The technique presented was based on restructuring the surface of the carbide tool by selective regrowth of carbide grains at the surface to obtain large anchor points for the diamond film. At the same time the cobalt concentration at the surface was reduced so that the potential for graphite formation between the diamond and the substrate was much lower. His conclusion was that this system completely solves the adhesion problem and that other film properties could now be optimized.

Testing for adequate adhesion of diamond to carbide inserts is always problematic and M. Drory of Crystallume presented data to illustrate the technique of brale indentation to measure adhesion of diamond to titanium alloy substrates. His results clearly showed the value of the technique for use on brittle films over relatively ductile substrates. In these cases consistent quantitative data was obtained. Results of indents on tungsten carbide substrates were not presented but Drory stated that correlation between indent measurements and tool machining performance was not straightforward in this case and would require more analysis and testing.

Drory’s presentation and several others discussed the difficulty of measuring adhesion using contact type techniques such as indentation or scratch tests. V. Sarin of Boston University offered additional evidence concerning the difficulty of using contact type measurement techniques and as to why these techniques are not applicable to brittle films on brittle substrates. He then discussed a noncontact measurement technique using a compression test to measure adhesion of diamond films to silicon nitride substrates. Using a three dimensional numerical model he was able to correlate the load force to the direct characteristics of adhesion such as debonding shear stress and elastic energy of delamination. This test would require specific substrate shapes but appeared to be directly applicable to WC-Co substrates.

The fourth session contained presentations from NIST researchers. S. Jahanmir gave a detailed discussion of a round robin set of measurements on ceramic grinding with diamond wheels. He demonstrated how statistical design of the experiment conducted prior to the measurements can allow a systematic evaluation of the data. A. Feldman reviewed the results of a NIST project for developing nondestructive evaluation (NDE) techniques to evaluate diamond film adhesion. He showed that a thermal wave image of an indent in a diamond coated WC specimen may reveal large areas of delamination not observable by SEM or optical inspection alone. Indentation testing is frequently used to evaluate diamond film adhesion.

The symposium concluded with a discussion of the need for standards and guides for users of diamond coated cutting tools, lead by A.P. Malshe. While protocols for machining with diamond coated cutting tools would be useful, the large range of machining conditions that exist would make this a formidable task.

## 3. Working Group on Standardizing Thermal Conductivity Measurement

A new round robin for measuring the thermal conductivity is to be held by consensus of the participants. A new round robin is to be conducted because of the relatively large variations in measured values reported in the previous round robin.[1] The specimens had large inhomogeneities. It is believed that the different ways the various measurement methods averaged over the inhomogeneities contributed to the variations observed. Discussions were held to finalize the details of the second round-robin. Many suggestions that had been faxed or e-mailed prior to the meeting were presented for discussion resulting in a new plan.
